# How Work Impairments and Reduced Work Ability are Associated with Health Care Use in Workers with Musculoskeletal Disorders, Cardiovascular Disorders or Mental Disorders

**DOI:** 10.1007/s10926-013-9492-3

**Published:** 2014-01-04

**Authors:** Kerstin G. Reeuwijk, Suzan J. W. Robroek, Leona Hakkaart, Alex Burdorf

**Affiliations:** 1 Department of Public Health, Erasmus Medical Center, PO Box 2040, 3000 CA Rotterdam, The Netherlands; 2 Institute of Health Policy and Management (iBMG), Institute for Medical Technology Assessment, Erasmus University, PO Box 1738, 3000 DR Rotterdam, The Netherlands

**Keywords:** Work ability, Work impairments, Health care utilization, Musculoskeletal disease, Cardiovascular disease, Mental disorders

## Abstract

*Purpose* the aim of this study was to explore how work impairments and work ability are associated with health care use by workers with musculoskeletal disorders (MSD), cardiovascular disorders (CVD), or mental disorders (MD). *Methods* in this cross-sectional study, subjects with MSD (n = 2,074), CVD (n = 714), and MD (n = 443) were selected among health care workers in 12 Dutch organizations. Using an online questionnaire, data were collected on individual characteristics, health behaviors, work impairments, work ability, and consultation of a general practitioner (GP), physiotherapist, specialist, or psychologist in the past year. Univariate and multivariate logistic regression analyses were performed to explore the associations of work impairments and work ability with health care use. *Results* lower work ability was associated with a higher likelihood of consulting any health care provider among workers with common disorders (OR 1.05–1.45). Among workers with MSD work impairments increased the likelihood of consulting a GP (OR 1.55), specialist (OR 2.05), and physical therapist (OR 1.98). Among workers with CVD work impairments increased the likelihood of consulting a specialist (OR 1.94) and physical therapist (OR 2.73). Among workers with MD work impairments increased the likelihood of consulting a specialist (OR 1.79) and psychologist (OR 1.82). *Conclusion* work impairments and reduced work ability were associated with health care use among workers with MSD, CVD, or MD. These findings suggest that addressing work-related problems in workers with common disorders may contribute in reducing health care needs.

## Introduction

Health care use and subsequent costs are rising in Western countries [1, 2]. In the Netherlands, health care expenditures have almost doubled in the past decade, which can only partly be attributed to aging of the population [3]. The total health care costs were approximately 74 billion euro in 2007 (for 16 million inhabitants) of which cardiovascular disorders (CVD) accounted for 9.3 %, mental disorders (MD) excluding dementia and mental disabilities for 9.1 %, and musculoskeletal disorders (MSD) for 6.6 % [4–6]. In order to retain an affordable health care it is important to identify modifiable risk factors for health care use which may be targeted in interventions.

To date, numerous studies have reported that health care use is associated with the presence and severity of diseases [7–9]. Similarly, the role of individual characteristics [9–11] and lifestyle [12–14] on health care use among those with health complaints is well studied. Since the majority of adults are engaged in paid employment, it is of particular interest to evaluate how well workers with health problems cope with demands at work. It has been well documented that poor health is an important predictor of productivity loss at work, sickness absence, decreased work ability, and exit from work [15–19].

Surprisingly, less is known about how the interplay between work demands and perceived health problems may influence health care use. Several studies have shown that a decreased work ability, defined as a person’s physical and mental ability to cope with the demands of work [20], is associated with increased sickness absence (RR 3.58), productivity loss at work (OR 4.08–5.54), and disability pension (OR 34.16) [19, 21–23]. Previous research has also suggested an association between adverse physical and psychosocial work-related factors and higher health care use among people with MSD [24, 25]. Similarly, in the general working population, psychosocial risk factors at work were found to prompt visiting a general practitioner (GP) or specialist [26].

Although some studies have identified the influence of work-related risk factors for increased health care use, the importance of work impairments due to health problems and the work ability on health care use have barely been studied. While previous studies often focused on health care use for one particular disease, this study incorporated workers with three common disorders (physical as well as mental) which account for a substantial proportion of health care expenditures.

This present study aims to investigate the association of work impairments and work ability with health care use among workers with MSD, CVD, and MD.

## Methods

### Study Sample and Data Collection

The study population consisted of workers from 12 health care organizations in Limburg, the Netherlands. These organizations comprised a hospital (n = 1), a nursing home (n = 1), homes for physically or mentally handicapped persons (n = 4), mental health care organizations (n = 4), a home care organization (n = 1), and a maternal care organization (n = 1). These organizations had commissioned an occupational health organization to launch a program to investigate the sustainable employability of their workforce. As part of this program, an online questionnaire survey was conducted on health, health care use, work ability, and work impairments. Each participant was notified at the start of the questionnaire that the information would be used for scientific purposes and that filling out the questionnaire was considered as informed consent. It was ensured by the occupational health organization that all potentially identifying information, such as names of workers, company or department, was removed from their database before data transfer to Erasmus MC, who guaranteed strict confidentiality of individual, non-coded information. This procedure is in agreement with the Dutch Code of Conduct for health research [27].

All workers from the participating organizations (n = 9,516) were invited by the occupational health organization by regular mail or email, which provided workers with an individualized password, to fill out the questionnaire on a secure website. Data collection took place between September 2011 and July 2012. The response ranged from 39 to 95 % across organizations. Total response was 55 % (n = 5,217), nine workers were excluded from the analysis because of incomplete data. Thus, complete data on health care use, work impairments, work ability, lifestyle, and individual characteristics were available for 5,208 workers.

In the questionnaire each respondent indicated on a list of 13 disorders (accident, MSD, CVD respiratory disorder, MD neurological disorder, genitourinary disorder, endocrine disorder, skin disorder, tumors, digestive system disorder, blood disorder, heritable disorder) whether they had a disorder that was diagnosed by a physician. In the current study we selected the two disorders with the highest prevalence (MSD with 2074 cases and CVD with 714 cases) and one disorder with a moderate prevalence but high health care use (MD with 443 cases).This selection covers 3 out of 4 chronic diseases with the highest burden of disease [28]. Some respondents had a combination of these disorders and they were considered in multiple categories.

### Measures

#### Individual Characteristics, Health, and Lifestyle-Related Factors

Information on gender, age, and education was collected. Age in years was categorized into four groups: <30, 30–40, 40–50, and ≥50 years. Education was assessed by the highest degree obtained and classified into three groups, i.e. high (higher vocational schooling or university), intermediate (higher secondary schooling or intermediate vocational schooling), and low (primary school, lower and intermediate secondary schooling, or lower vocational schooling).

The presence of disorders was assessed with the third question of the work ability index (WAI) [20]. This question is a limitative list of 13 broad categories of currently present diseases, diagnosed by a physician, with dichotomous answers. This list of self-reported diagnosed disorders was used to select workers with MSD, CVD or MD, as well as to assess multimorbidity. Multimorbidity was considered present when a worker reported more than one disorder.

Information on smoking, physical activity, and body mass index (BMI) was collected. Smoking was assessed using one question ‘Do you smoke?’ (yes/no). Leisure-time physical activity was assessed on the basis of one question if workers were daily physically active for at least 30 min during leisure-time (yes/no). Self-reported height in meters and weight in kilograms were assessed, and were used to calculate BMI (kg/m^2^). Three BMI categories were defined: normal (BMI < 25 kg/m^2^), overweight (BMI between 25 and 30 kg/m^2^), and obese (BMI ≥ 30 kg/m^2^) [29].

#### Impairments and Work Ability

Work impairments were measured using the fourth question of the WAI list [20]. This question addressed current functional limitations due to health problems, based on an ordinal scale. Answers were classified into three categories: no impairments (no impairments or no disorders, diseases or complaints); moderate impairments (able to perform one’s job, but with some impairments or sometimes have to adjust working pace or the way of working); and severe impairments (individuals have often adjusted work pace and activities, or are capable only of part-time work, or are unable to work at all).

Work ability was measured with the first question of the WAI [20]. This question rated a worker’s current work ability relative to the best work ability during their life on an 11-point scale ranging from zero (unable to work) to ten (current work ability equals best work ability ever). Lower work ability was used as a continuous variable, expressed by the decrease in work ability relative to the maximum score of ten.

#### Health Care Use

Self-reported information on consulting a health care provider in the previous 12 months was measured. Six dichotomous variables (yes/no) indicated whether a GP, specialist, physical therapist, psychologist, psychiatrist, or other health care provider had been consulted during the past 12 months. Due to similarities in underlying health problems, visiting a psychologist and psychiatrist were merged into one group. The group of ‘other health care providers’ was left out due to the large variety in health providers, like dentist or gynecologist, which were not considered relevant for the current study. The questions on health care use referred to health problems in general and were not specific for distinguished common disorders.

### Analysis

For the main variables, descriptive statistics were generated, i.e. frequencies and percentages for dichotomous and categorical variables, and means and standard deviations for continuous variables. Logistic regression analyses were used to estimate among workers with specific disorders the associations between work impairments and lower work ability with health care use (consulting a GP, specialist, physical therapist, and psychologist or psychiatrist). Type of care was not mutually exclusive. All multivariate analyses were adjusted for individual characteristics (gender, age, and education level), multimorbidity, and lifestyle-related factors (smoking, physical activity, and BMI). The odds ratios (OR) with corresponding 95 % confidence intervals (95 % CI) were calculated as the measure of association. A *p* value below 0.05 was considered statistically significant. All analyses were conducted with the Statistical Package for Social Sciences (SPSS) version 20.0 for Windows (IBM Software, Chicago).

## Results

**Fig. 1:**
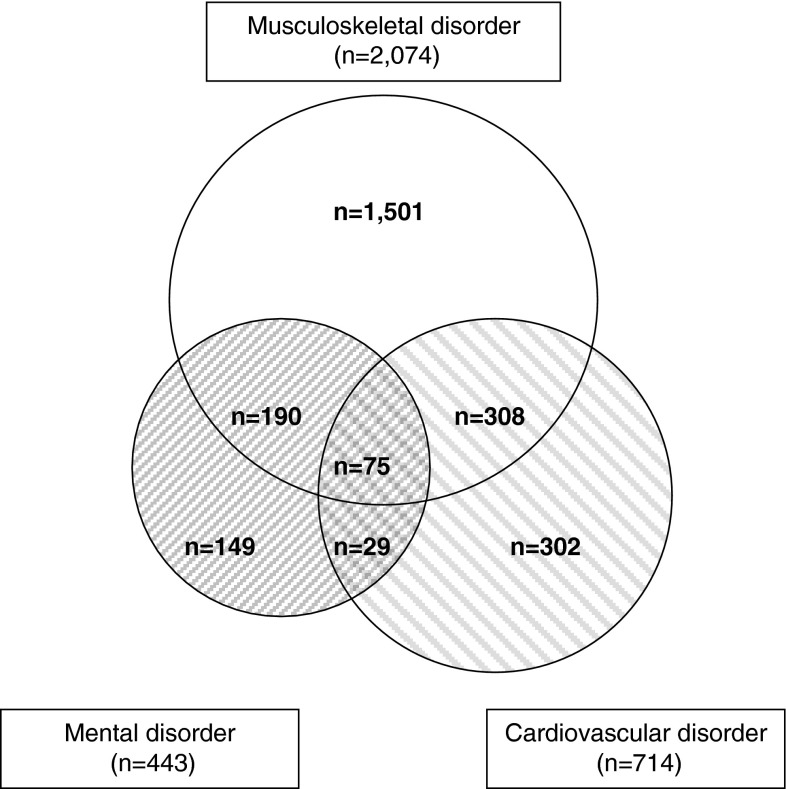
*Venn diagram* for the overlap between MSD (n = 2,074), CVD (n = 714), and MD (n = 443) within health care workers from 12 Dutch organizations

**Fig. 2:**
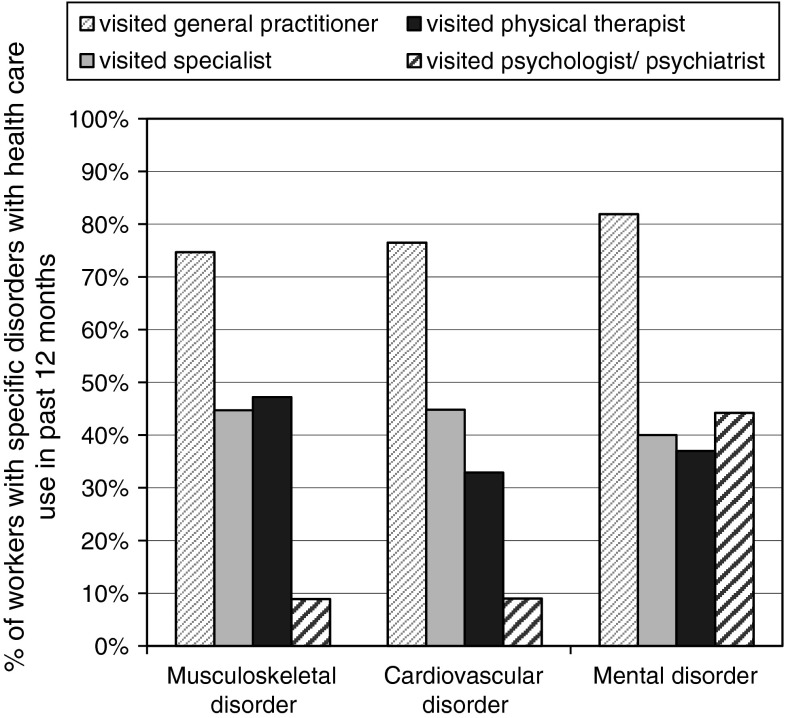
Prevalence of health care use (GP, specialist, physical therapist, and psychologist/psychiatrist) among health care workers with MSD (n = 2,074), CVD (n = 714), and MD (n = 443), from 12 Dutch organizations

The characteristics of the respondents with MSD, CVD, and MD are presented in Table 1. The majority of the total study population (n = 5,208) was female (80 %) and age ranged from 17 to 66 years, with a mean of 43.2 years (± 11.4 years). Men (response 53 %) and women (response 55 %) were equally likely to fill in the questionnaire. Workers younger than 20 years of age (response 30 %) were less likely to fill in the questionnaire than older workers (response 55 %). Forty percent of the workers had MSD, 14 % CVD, and 9 % MD. Severe work impairments were present in 11 % of workers and 36 % had moderate impairments. Work ability was reduced on average by 2.1 points (±1.6). There was a moderate association between work impairments and work ability (Pearson r 0.38). Some workers had a combination of the three disorders of interest (MSD, CVD, MD), the overlap in this study between these disorders is shown in Fig. 1. Multimorbidity with other disorders than MSD, CVD, or MD was also possible. Overall, multimorbidity was present in 69 % of those with MSD, 75 % of those with CVD, and 85 % of those with MD. Figure 2 shows that the GP was the most consulted health care provider in the previous 12 months, with 75 % among workers with MSD, 76 % of those with CVD, and 82 % of those with MD. Health care use of all providers was significantly different between the three disorders.

**Table 1:** Population characteristics of health care workers with MSD (n = 2,074), CVD (n = 714), and MD (n = 443) from 12 Dutch organizations

	MSD (n = 2,074) n (%)	CVD (n = 714) n (%)	MD (n = 443) n (%)
Individual characteristics			
Age (years)			
<30	239 (11.5)	21 (2.9)	59 (13.3)
30–40	361 (17.4)	59 (8.3)	89 (20.1)
40–50	574 (27.7)	177 (24.8)	115 (26.0)
≥50	900 (43.4)	457 (64.0)	180 (40.6)
Gender, female	1,694 (81.7)	517 (72.4)	348 (78.6)
Education			
Low	245 (11.8)	118 (16.5)	49 (11.1)
Intermediate	1,164 (56.1)	333 (46.6)	243 (54.9)
High	665 (32.1)	263 (36.8)	151 (34.1)
Work-related factors			
Work impairments			
No	517 (24.9)	262 (36.7)	83 (18.7)
Moderate	1,151 (55.5)	322 (45.1)	219 (49.4)
Severe	406 (19.6)	130 (18.2)	141 (31.8)
Reduced work ability^a^, mean (sd)	2.5 (1.7)	2.3 (1.5)	3.4 (2.0)
Multimorbidity			
More than one disorder diagnosed	1,428 (68.9)	534 (74.8)	378 (85.3)
Lifestyle			
Current smoker	483 (23.3)	137 (19.2)	127 (28.7)
Insufficient physical activity in leisure time	710 (34.2)	240 (33.6)	157 (35.4)
BMI			
Normal weight	1,032 (49.8)	254 (35.6)	222 (50.1)
Overweight	737 (35.5)	305 (42.7)	145 (32.7)
Obese	305 (14.7)	155 (21.7)	76 (17.2)

^a^Range 0–10, zero work ability in the best period, ten not able to work at all

*MSD* musculoskeletal disorder, *CVD* cardiovascular disorder, *MD* mental disorder, *n* number of workers, *sd* standard deviation, *BMI* body mass index

### Determinants of Health Care Use

#### Musculoskeletal Disorder

Both work impairments and work ability were associated with consultation of any health care provider. The univariate analyses showed that workers with a lower work ability were more likely to consult a health care provider than workers with excellent work ability [ORs per 10 % lower work ability ranged between 1.12 (95 %CI 1.06–1.18) for physical therapist and 1.37 (95 % CI 1.27–1.48) for psychologist/psychiatrist (data not shown)]. In the multivariate analyses including both work impairments and work ability and all potential confounders (individual characteristics, multimorbidity, and lifestyle-related factors) the strength of the association between work ability and health care use reduced slightly, ranging between 2 % decrease for psychologist/psychiatrist (OR 1.37–OR 1.35) and 8 % decrease for visiting a specialist (OR 1.20–OR 1.10). All associations remained statistically significant for all health care providers, except for physical therapists (OR 1.05, 95 %CI 0.99–1.12) (Table 2). Workers with moderate or severe work impairments were statistically significantly more likely to consult a GP, specialist, or physical therapist than workers who experienced no impairments (Table 2). A statistically significant trend was observed for increasing severity of work impairments and a higher likelihood to consult a specialist or physical therapist.

**Table 2:** Multivariate logistic regression analyses with OR and 95 % confidence intervals for work impairments, lower work ability and health care use among health care workers with MSD (n = 2,074), CVD (n = 714), or MD (n = 443), from 12 Dutch organizations

	GP	Specialist	Physiotherapist	Psychologist
	OR (95 % CI)^b^	OR (95 % CI)^b^	OR (95 % CI)^b^	OR (95 % CI)^b^
MSD (n = 2,074)				
Work impairments				
no	1	1	1	1
moderate	1.64 (1.29–2.08)*	1.19 (0.94–1.49)	1.33 (1.07–1.66)*	1.32 (0.84–2.09)
severe	1.55 (1.09–2.21)*	2.05 (1.50–2.80)*	1.98 (1.46–2.68)*	1.34 (0.76–2.37)
Lower work ability^a^	1.10 (1.02–1.18)*	1.10 (1.04–1.17)*	1.05 (0.99–1.12)†	1.35 (1.23–1.47)*
CVD (n = 714)				
Work impairments				
no	1	1	1	1
moderate	1.23 (0.80–1.90)	1.52 (1.04–2.23)*	2.16 (1.41–3.32)*	1.76 (0.79–3.93)
severe	0.89 (0.50–1.60)	1.94 (1.17–3.22)*	2.73 (1.58–4.70)*	1.61 (0.62–4.22)
Lower work ability^a^	1.23 (1.06–1.42)*	1.11 (0.99–1.25)†	1.08 (0.96–1.21)	1.45 (1.22–1.72)*
MD (n = 443)				
Work impairments				
no	1	1	1	1
moderate	1.55 (0.80–3.02)	1.25 (0.69–2.25)	1.45 (0.78–2.67)	1.55 (0.86–2.78)
severe	0.98 (0.46–2.07)	1.79 (0.92–3.48)†	1.55 (0.78–3.07)	1.82 (0.93–3.54)†
Lower work ability^a^	1.23 (1.06–1.43)*	0.99 (0.89–1.11)	1.11 (1.00–1.24)†	1.13 (1.01–1.26)*

^a^Range 0–10, zero work ability in the best period, ten unable to work at all. ^b ^adjusted for individual factors (age, gender, education), multimorbidity, and lifestyle-related factors (smoking, physical activity, BMI). *p value <0.05. †*p* value <0.10

*GP* general practitioner, *OR* odds ratio, *95* *%CI* 95 % confidence interval, *n* number of workers, *MSD* musculoskeletal disorder, *CVD* cardiovascular disorder, *MD* mental disorder

#### Cardiovascular Disorder

Both work impairments and lower work ability were associated with a higher likelihood of consulting a health care provider. Lower work ability was associated with a higher likelihood of consulting all health care providers in the univariate analyses [ORs per 10 % decrease in work ability ranging between 1.23 (95 % CI 1.11–1.36) for specialists and 1.51 (95 % CI 1.30–1.74) for psychologists (data not shown)]. In the multivariate analyses, the strength of the association between work ability and health care use reduced, ranging between 4 % decrease for visiting a GP (OR 1.28–OR 1.23) and 13 % decrease for visiting a physical therapist (OR 1.24–OR 1.08). The associations between work impairments, work ability, and consulting a GP or a psychologist, remained statistically significant in the multivariate analysis (Table 2). Workers with severe work impairments were more likely to consult a specialist (OR 1.94, 95 %CI 1.17–3.22) or physical therapist (OR 2.73, 95 %CI 1.58–4.70) than workers without work impairments (Table 2).

#### Mental Disorder

Both work impairments and work ability were associated with a higher likelihood of consulting a health care provider. Workers with a lower work ability were statistically significantly more likely to consult a GP, physical therapist, and psychologist than workers with excellent work ability [OR per 10 % decrease in work ability ranging from 1.13 (95 % CI 1.03–1.25) for physical therapists and 1.22 (95 % CI 1.06–1.40) for GPs (data not shown)]. In the multivariate analyses, adjustment for confounders changed associations between work ability and health care use with less than 5 %. Similar patterns were observed in the multivariate and univariate analyses for the associations between work impairments and consultation of a health care provider (Table 2). Although not statistically significant at *p* < 0.05, the associations between severe work impairments and visiting a specialist (OR 1.79, 95 %CI 0.92–3.48) or a psychologist (OR 1.82, 95 %CI 0.93–3.54) indicated that workers with severe work impairments were more likely to consult a specialist or a psychologist than workers without impairments (Table 2).

## Discussion

Work impairments were associated with health care use among health care workers with MSD, CVD, or MD. Similarly, workers with lower work ability were more likely to consult a GP, a specialist, a physiotherapist, and a psychologist. Despite the moderate correlation between work impairments and work ability, the results of the current study suggest that a lower work ability as well as perceived impairments at work might be a prompt for workers to seek health care.

### Health Care Use Among Workers with MSD, CVD, and MD

Health care use seemed relatively high in comparison with other studies. Among workers with MSD, almost 75 % visited a GP, 45 % a specialist, and 48 % a physical therapist in the past 12 months. However, one has to bear in mind that the health care use was not limited to care seeking for a specific disorder. The method of population attributable fraction may be used to attribute health care use to the presence of a specific disorder, based on the prevalence of the specific disorder and the likelihood of health care use among workers with that disorder relative to workers without that disorder [30]. We observed proportions in line with other studies. For example, Molano et al. [31] found that 44 % of scaffolders with low back pain visited a GP. This is comparable with the population attributable fraction of 42 % in our study population. Similarly, Ikonen et al. [32] found that 46 % of male workers, and 51 % of female workers with physician-diagnosed MSD visited an occupational health physician. As for MD, our finding that 44 % visited a psychologist or psychiatrist was also relatively high when compared with previous studies [33, 34]. They reported that 21–25 % of the individuals with a psychiatric disorder reported the use of mental health services [33, 34], which is almost twice as low as what we found. On the other hand, the same studies found that 83–91 % of these subjects visited a primary care physician, which is in line with our finding that 82 % visited a GP. The relative high numbers of health care use in our study population may be explained by the fact that our population consisted of workers in the health care sector, hence, they likely know how to access health care better than workers from other sectors. More than half of our study population had an intermediate education level, indicating that the majority of our sample probably consists of nursing personnel, and assisting personnel (e.g. receptionists, administrative workers), rather than physicians. Only limited evidence is present about health care use among nursing personnel. One study found that nurses use health care less often than the general population [35]. Information about health care use of other workers within the health care sector is lacking.

The GP was the most commonly visited health care provider in this study. This is due to the fact that the GP serves as gate keeper in the Dutch health care system. Hence, the GP is often the first health care provider to be consulted by individuals before having access to other services like specialist care. In the Netherlands, the physical therapist and psychologist can be visited without referral of the GP. However, their services are not always fully covered by the health insurance, unlike the GP visits [36]. Therefore, individuals may be less likely to consult these health providers compared with a GP.

### Work Impairments Among Workers with MSD, CVD, and MD

A large part of the respondents with MSD, CVD, and MD had moderate or severe work impairments (75.1, 63.3, and 81.3 % respectively). A recent study among employees from a large Dutch railway company reported among workers with musculoskeletal complaints about 50 % experienced work impairments due to these complaints [37]. Comparable results were reported for persons with heart disease (48 %), major depression (45 %), and generalized anxiety disorder (54 %) [38]. The higher occurrence of impairments in our study population is due to the fact that we could not distinguish between impairments due to a specific disorder and impairments due to the considerable multimorbidity that was adjusted for in the analysis. Since mutual adjustment of MSD, CVD, and MD did not change the results presented in Table 2, the influence of work impairments is not limited to those workers who had a combination of MSD, CVD, and MD.

Previous studies showed that sickness absence [39] and productivity loss at work [19] was also relatively common in people with MSD, CVD, or MD. Leijten et al. [39] reported that among workers with MD about 70 % had at least 1 day of sickness absence in the previous year, and among workers with MSD and CVD these figures were 55 and 52 %, respectively.

### Work Impairments, Work Ability, and Health Care Use

Earlier studies have shown that a reduced WAI predicts disability benefit [22], reduced work productivity, and sickness absence [21, 23]. In the current study a reduced work ability was also associated with health care use.

Several recently published studies also explored the association between work ability in the general workforce (i.e. not within workers with specific disorders) and health care use. A longitudinal study from Germany [40] and two Scandinavian studies [32, 41] observed an association between a poor work ability as measured with the WAI and health care use. A limitation of these studies is that the measure of work ability, the WAI, includes several questions on presence of health problems and experienced functional limitations, which in itself may explain health care use [7–9]. In the present study the concept of work ability was measured by a single question, independent from health status. Our results showed that a lower work ability was associated with an increased likelihood of health care use. The OR for the associations between work ability and health care use were presented per 10 % lower work ability score in this study. Hence, for example when work ability is strongly reduced to a score of five (instead of the maximum of ten) the OR for visiting a psychologist among respondents with CVD accumulates from 1.45 to 6.36 and indicates a substantially increased likelihood of consulting a health care provider.

Among workers with common disorders the perceived impairments while performing their regular activities at work prompted seeking health care. For most disorders the severity of impairments did seem to increase health care use, but only for MSD a statistically significant trend was observed for degree of severity and likelihood of having visited a specialist or physiotherapist. Thus, although our study population contained many workers with common disorders, the study lacked some power to disentangle determinants of specific referral patterns.

### Work Impairment, Work Ability, and Health Care Use in MSD, CVD, and MD

To our knowledge this is one of the first studies exploring the association between work impairments, work ability, and health care use within specific disorder groups. For mental as well as physical disorders, workers with work impairments and workers with a lower work ability were more likely to visit a health care professional. These results suggest that focusing on work impairments and keeping good work ability may be important for future interventions to reduce the need for health care use among workers with common mental or physical disorders.

### Strength and Limitations

A strength of the present study is the large study population. However, some limitations need to be addressed. Firstly, we had no information on severity of MSD, CVD, and MD and therefore it could not be established how disease severity could have influenced the observed associations between work impairments and health care use. Several studies have shown that severity of disease is associated with work impairments [42, 43] and, thus, it may be hypothesized that both disease severity and impairments may prompt health care use. It may even be considered that work impairments partly mediates the association between disease severity and health care use. In these explanations both disease severity and work impairments are targets for intervention, whereby the exact interplay between disease severity, work impairments, and work conditions will guide the actual content of effective interventions in occupational groups. Secondly, the cross-sectional design does not permit the establishment of causal relationships. However, the findings suggest that attention for chronic health problems as well as attention for impairments at work due to these health problems is likely to be relevant. Thirdly, results of this study were based on dichotomous data of consulting a health provider and frequency of use was not taken into account due to lack of precise information on frequency of health care use. Consequently, we were also unable to make statements about subsequent health care expenditures. Fourthly, health care use, work impairments, and work ability were all self-reported and thus vulnerable to recall bias [44, 45]. However, self-reported work ability is a widely used measure in the field of occupational health and it has shown to be a predictor for long-term sick leave, productivity loss, and disability benefit [21–23]. Lastly, the group ‘other health provider’ was left out of this study. Some respondents indicated that they had visited a specialist, such as dermatologist, gynecologist, or rheumatologist. In a sensitivity analysis we included these care providers in the category ‘specialist’, but the results changed very little due to a low frequency of care seeking.

Since the study population consisted of a selective group of workers with a paid job in the health care sector, results may differ in other sectors. Around 80 % of the study population was female, which is representative for workers in the health care sector [46, 47].

## Conclusion

Work impairments and reduced work ability were associated with health care use among health care workers with MSD, CVD, or MD. These findings suggest that addressing work-related problems in workers with common disorders may contribute in reducing health care needs.
